# Molecular mechanism of inhibitory effects of bovine lactoferrin on the growth of oral squamous cell carcinoma

**DOI:** 10.1371/journal.pone.0191683

**Published:** 2018-01-30

**Authors:** Chanbora Chea, Mutsumi Miyauchi, Toshihiro Inubushi, Nurina Febriyanti Ayuningtyas, Ajiravudh Subarnbhesaj, Phuong Thao Nguyen, Madhu Shrestha, Sivmeng Haing, Kouji Ohta, Takashi Takata

**Affiliations:** 1 Department of Oral & Maxillofacial Pathobiology, Graduate School of Biomedical and Health Sciences, Hiroshima University, Hiroshima, Japan; 2 Sanford Burnham Prebys Medical Research Institute, San Diego California, United States of America; 3 Department of Oral Diagnostic Science, Faculty of Dentistry, Khon Kaen University, Khon Kaen, Thailand; 4 Department of Global Dental Medicine & Molecular Oncology, Graduate School of Biomedical and Health Sciences, Hiroshima University, Hiroshima, Japan; 5 Department of Oral and Maxillofacial Surgery, Division of Cervico-Gnathostomatology, Graduate School of Biomedical and Health Sciences, Hiroshima University, Hiroshima, Japan; Charles P. Darby Children's Research Institute, 173 Ashley Avenue, Charleston, SC 29425, USA, UNITED STATES

## Abstract

**Background:**

Lactoferrin (LF), a member of the transferrin family, recently has been demonstrated to have anticancer effects on various cancers including oral squamous cell carcinoma (OSCC). However, little is known about the underlying mechanisms of its effects on OSCC. Therefore, we aimed to investigate the mechanism of the suppressive effects of bovine LF (bLF) on the growth of OSCC cells.

**Methods:**

In the current study, HSC2, HSC3, HSC4 and normal human oral keratinocytes (RT7) cell lines were tested with bLF 1, 10, and 100 μg/ml. The effects and detail mechanisms of bLF on proliferation and apoptosis of cells were investigated using flow cytometry and western blotting.

**Results:**

We found that bLF (1, 10, and 100 μg/ml) induced activation of p53, a tumor suppressor gene, is associated with the induction of cell cycle arrest in G1/S phase and apoptosis in OSCC. Moreover, bLF downregulated the phosphorylation of Akt and activated suppressor of cytokine signaling 3 (SOCS3), thereby attenuating multiple signaling pathways including mTOR/S6K and JAK/STAT3. Interestingly, we revealed that bLF exerted its effect selectively against HSC3 but not on RT7 via different effects on the phosphorylation status of NF-κB and Akt.

**Conclusion:**

This is the first report showing that bLF selectively suppresses proliferation through mTOR/S6K and JAK/STAT3 pathways and induction of apoptosis in OSCC. This study provides important new findings, which might be useful in the prevention and treatment of OSCC.

## Introduction

Anti-cancer drugs derived from natural compounds are highly safe for the treatment of cancers. Lactoferrin (LF), an 80-kDa member of the transferrin family of iron binding glycoproteins [1], is naturally produced by epithelial cells [2] and found in external secretions mainly in milk [3]. Diverse biological functions of LF such as anti-inflammatory [4], anti-viral [5], anti-bacterial [6], and anti-tumor [7–10] have been reported. Interestingly, for many years, LF and its derivatives have been proposed for cancer therapy due to their multiple tumor-targeting and high cytological effects on cancer cells [11–13].

Inhibition of cell cycle progression in cancer cells is one of the main mechanisms by which LF may inhibit cancer growth. Recent studies have shown that LF directly arrested cell cycle in G1/S transition in breast and head and neck cancers both *in vitro* and *in vivo* via regulation of cell cycle-associated proteins including Akt, p21, p19, p27, Cdk2, cyclin E, Cdk4, and cyclin D1 [10,14–16].

In addition, LF and its derivative peptides were also reported to induce apoptosis in stomach cancer by the activation of caspase 9 [17]; in breast cancer via increase in DNA fragmentation accompanied by chromatin condensation and nuclear fragmentation [18]; and in leukemia through activation of caspase 2, 9, and 3 [19–21].

The mammalian target of rapamycin (mTOR), a serine/threonine kinase, is activated by Akt signaling pathway and sequentially phosphorylates S6 kinase (S6K) [22] to modulate cellular growth. mTOR has recently been considered as an attractive target in cancer therapy [23,24]. Suppressor of cytokine signaling 3 (SOCS3) is a negative feedback regulator of growth signaling [25]. Recently, SOCS3 has been shown to deregulate Janus kinase and signal transducer and activator of transcription (JAK2/STAT3) signaling [26] via its interaction with receptor JAK2 through the SH2 domain [27]. In addition, restoration of SOCS3 expression attenuated STAT3 activation, induced apoptosis, and suppressed tumor cell growth in lung cancer [28].

LF and its derivatives are shown to reduce cancer cell growth; however, there is a limited understanding about the mechanisms involved in LF-mediated growth inhibition in oral squamous cell carcinoma (OSCC). Hence, investigating the effects of LF on cell proliferation pathways such as mTOR and JAK2/STAT3/SOCS3 can provide new insights of its action. Therefore, we comprehensively investigated the biological effects and detail mechanisms of bLF on growth of OSCC.

## Methods

### Reagents

Bovine lactoferrin (bLF) was procured from Sunstar Inc. (Osaka, Japan). Propidium Iodide (PI), LY294002, S3I-201, PF-4708671, and Caffeic acid (CAPE) were purchased from Sigma-Aldrich (MO, USA). PE Annexin V Apoptosis Detection Kit was obtained from BD Biosciences (CA, USA). Rapamycin was purchased from Santa Cruz Biotechnology (CA, USA).

### Bovine lactoferrin preparation

bLF was reconstituted in sterilized-distilled water at a concentration of 10 mg/ml. Reconstituted bLF was mixed homogenously by vortexing, filtered through 0.2 μm pore size filter, and stored at -20°C.

### Cell culture

The immortalized human oral keratinocyte cell line RT7 was maintained in Keratinocyte-SFM (Gibco BRL, Gaithersburg, MD, USA) supplemented with 25 μg/ml of bovine pituitary extract, 0.5 ng/ml of epidermal growth factor, 100 U/ml of penicillin, and 100 μg/ml of streptomycin. Human epithelial OSCC cell lines; HSC2, HSC3, and HSC4 were provided by Japanese Collection of Research Bioresources Cell Bank. These cell lines were cultured in RPMI-1640 (Nacalai Tesque, Inc., Tokyo, Japan) supplemented with 10% heat inactivated FBS (Biowest, France) and 100 U/ml of penicillin-streptomycin (Sigma-Aldrich, MO, USA). All cell lines were maintained in a humidified incubator at 37°C with 5% CO_2_.

### Proliferation assay

HSC2, HSC3, HSC4, and RT7 cells were plated in 24-well plate at a density of 3,000 cells per well. After 24 h of incubation, medium was replaced with bLF containing medium. Cells were then trypsinised and counted on days 1, 2, 4, and 6 using a cell counter (Coulter Electronic, UK). Each experiment was repeated at least 3 times in triplicate wells.

### Flow cytometry analysis

HSC3 cells (1 x 10^5^) were seeded in 10 cm dishes with or without bLF (1 μg/ml, 10 μg/ml, and 100 μg/ml) for 48 h at 37°C. Cells were then harvested and centrifuged at 3,000 rpm for 3 min. The pellets were washed three times with ice-cold phosphate buffered saline (PBS) (Nacalai Tesque Inc), re-suspended in 2 ml of ice-cold 70% ethanol and stored overnight at -20°C. After ethanol fixation, cells were washed twice with ice-cold PBS and centrifuged at 3,000 rpm. For cell cycle analysis, PI solution (10 μg/ml of PI and 100 μg/ml of RNase A in PBS) was added and samples were incubated at room temperature for 15 min prior to analysis. For apoptosis, cell pellets were suspended in 5 μl of PE-Annexin V and 5 μl of 7-AAD viability staining solution. Suspensions were gently mixed and incubated for 15 min at room temperature in the dark. After incubation, 400 μl of 1x Binding Buffer was added to each sample. Both cell cycle and apoptosis were measured using FACS Caliber flow cytometer (Becton-Dickinson, San Jose, CA).

### RT-PCR analysis

Total RNA was extracted from fresh HSC3 cells treated with and without bLF (1, 10, and 100 μg/ml) using RNeasy Mini Kit (Quiagen, K.K., Tokyo, Japan) according to manufacturer’s instructions. cDNA was reverse transcribed from 1μg of total RNA with ReverTra Dash Kit (Toyobo, Osaka, Japan). Total cDNA was amplified using Go Taq Green Master Mix (Promega, Madison, WI, USA), revolved using 1.5% agarose/TAE gels (Nacalai Tesque, Inc., Kyoto, Japan), and visualized by ethidium bromide staining. The following primer pair sequences were used: Forward, 5’-TGGCCACTCTTCAGCATCTC-3’, Reverse, 5’-AGCTGGGTGACTTTCTCATAGG-3’ for human SOCS3; Forward, 5’-AGCAAACGAGGCCTAAGTCA-3’, Reverse, 5’–GCTGCTTGTGCTGATGGTAA-3’ for human LRP1; Forward, 5’- GCATCCTGGGCTACACTGAG -3’, Reverse, 5’- TCCACCACCCTGTTGCTGTA -3’ for GAPDH.

### Western blotting

Cells were washed with ice-cold PBS and lysed in buffer containing 0.1% Triton X-100 (Roche, Castle Hill, Australia), 10 μg/ml L-1 chlor-3-(4-tosylamido)-4-Phenyl-2-butanon (TPCK), 1 mM DTT, 0.1 mM Na_3_VO_4_, 10 μg/ml L-1 chlor-3-(4-tosylamido)-7-amino-2-heptanon hydrochloride (TLCK), 0.1 mM leupeptin, and 50 μg/ml phenylmethylsulfonyl fluoride (PMSF) for 30 min. The lysates were centrifuged at 13,200 rpm for 20 min at 4°C. Supernatants were collected and protein concentration was determined by Bradford Protein Assay (Bio-Rad, Richmond, CA). The samples were then heated in 4 x Laemmli buffer at 100°C for 3 min. The proteins were resolved on 10% polyacrylamide gel (PAGE), and transferred to nitrocellulose membrane (Schleicher & Schuell, Dasse, Germany). The membranes were then blocked with 3% milk for 1 h at room temperature, and incubated with primary antibodies overnight at 4°C. The following antibodies were used: p-Akt (9271; 1:1,000; Cell Signaling Technology), pan-Akt (4691; 1:1,000; Cell Signaling Technology), p-NF-κB p65 (3033; 1:1,000; Cell Signaling Technology), NF-κB p65 (sc-109; 1:1,000; Santa Cruz Biotechnology), p-p53 (9281; 1:1,000; Cell Signaling Technology), p53 (9282; 1:1000; Cell Signaling Technology), p-mTOR (2971; 1:1,000; Cell Signaling Technology), mTOR (2983; 1:1,000; Cell Signaling Technology), p-S6K (9204; 1:1,000; Cell Signaling Technology), S6K (2708; 1:1,000; Cell Signaling Technology), SOCS3 (M20; 1:1,000), p-JAK2 (3771; 1:1,000; Cell Signaling Technology), JAK2 (3230; 1:1,000; Cell Signaling Technology), p-STAT3 (9133; 1:1,000; Cell Signaling Technology), STAT3 (9132; 1:1,000; Cell Signaling Technology), p-Bad (5284; 1:1,000; Cell Signaling Technology), p-Bcl2 (2827; 1:1,000; Cell Signaling Technology), cleaved caspase 9 (9505; 1:1,000; Cell Signaling Technology), cleaved caspase 3 (9664; 1:1,000; Cell Signaling Technology), cleaved caspase 6 (9761; 1:1,000; Cell Signaling Technology), cleaved caspase 7 (8438; 1:1,000; Cell Signaling Technology), BAD (695502; 1.0 μg/ml; Biolegend), Bcl2 (M0887; 1:1,000; Dako), LRP1 (2703–1; 1:7,000; Epitomics), p21 (sc-817; 1:1,000; Santa Cruz Biotechnology), cyclin D1 (M3642; 1:1,000; Dako), β-actin (A2228; 1:10,000; Sigma-Aldrich). Membranes were then incubated with appropriate secondary antibodies (1:1000) for 1 h at room temperature. The ECL western blotting detection system (GE Healthcare, UK) (Amersham, Piscataway, NJ, USA) was used for visualization of western blots.

### Statistical analysis

All numerical values reported represent mean values ± S.D. Statistical significance among groups was calculated using one-way ANOVA followed by Tukey’s post-hoc test. A value of *p < 0.05 and **p<0.01 were considered statistically significant.

## Results

### Bovine lactoferrin inhibited the proliferation of HSC2, HSC3, and HSC4 but not RT7 cells

**Fig 1:**
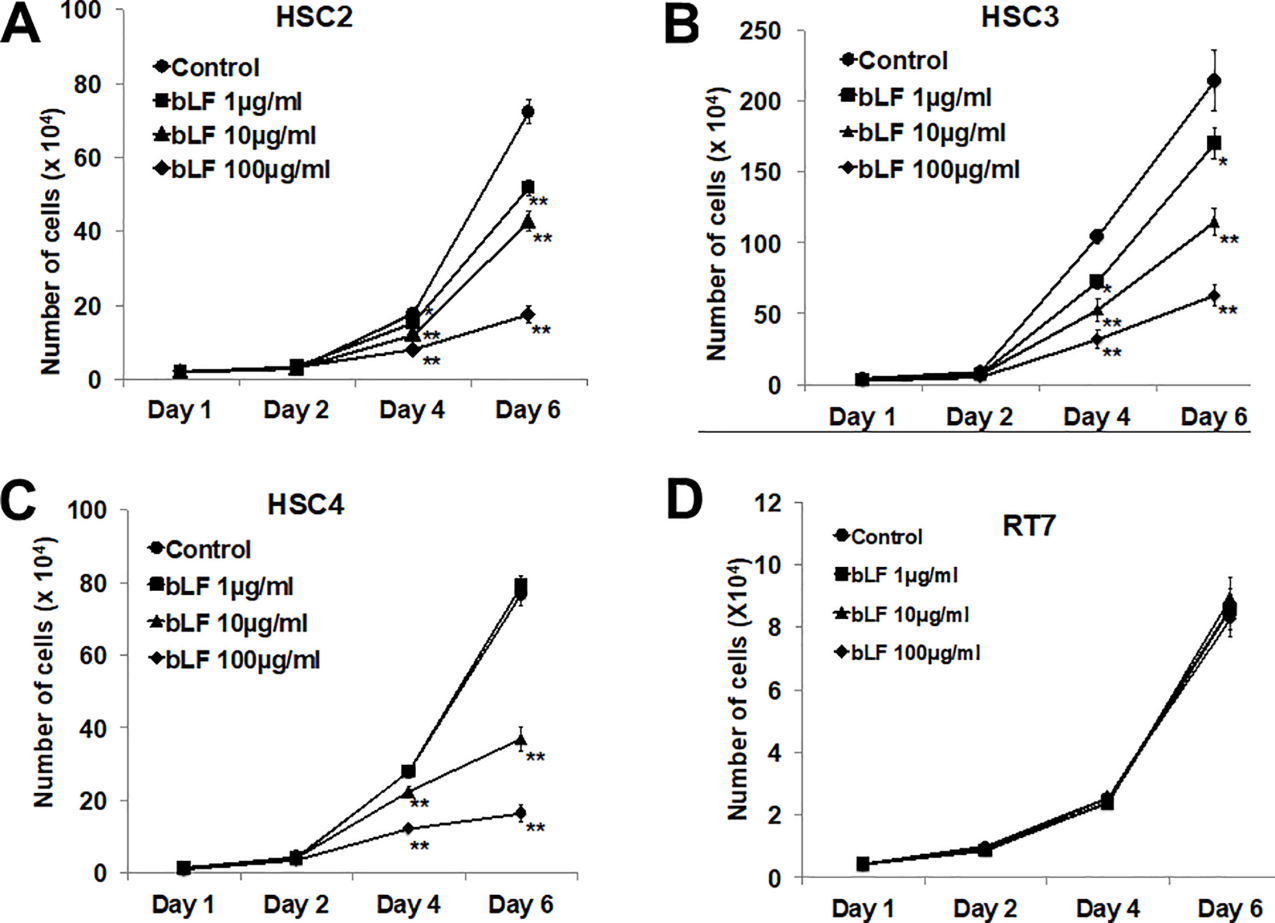
Effect of bovine lactoferrin on the proliferation of OSCC and normal mucosal cells. OSCC and RT7 cells were treated with different concentrations of bLF (1 μg/ml, 10 μg/ml, and 100 μg/ml) and cell number was counted on days 1, 2, 4 and 6. bLF significantly prohibited the cell proliferation of OSCC cell lines in a dose-dependent manner; (**A**) HSC2, (**B**) HSC3, and (**C**) HSC4. bLF did not affect the cell growth of RT7 cells (**D**). Data represented as mean ± S.D; * p < 0.05 and ** p < 0.01 *vs* control (0 μg/ml of bLF).

To investigate the effect of bLF on the proliferation of OSCC and normal mucosal epithelium, HSC2, HSC3, HSC4, and human oral keratinocyte (RT7) cell lines were used. In parallel, all cell lines were treated with different concentration of bLF (1, 10, and 100 μg/ml), and the number of cells was counted on days 1, 2, 4, and 6. The results indicated that bLF significantly suppressed the growth of HSC2, HSC3, and HSC4 cells in a dose-dependent manner (Fig 1A, 1B and 1C); however, it did not affect the growth of RT7 (Fig 1D). The results indicated that bLF inhibits the proliferation of OSCC cells but not the normal mucosal epithelial cells. To investigate the detail mechanisms of bLF action, HSC3 and RT7 cells were selected for further experiments.

### Bovine lactoferrin induced cell cycle arrest in HSC3 cells

**Fig 2:**
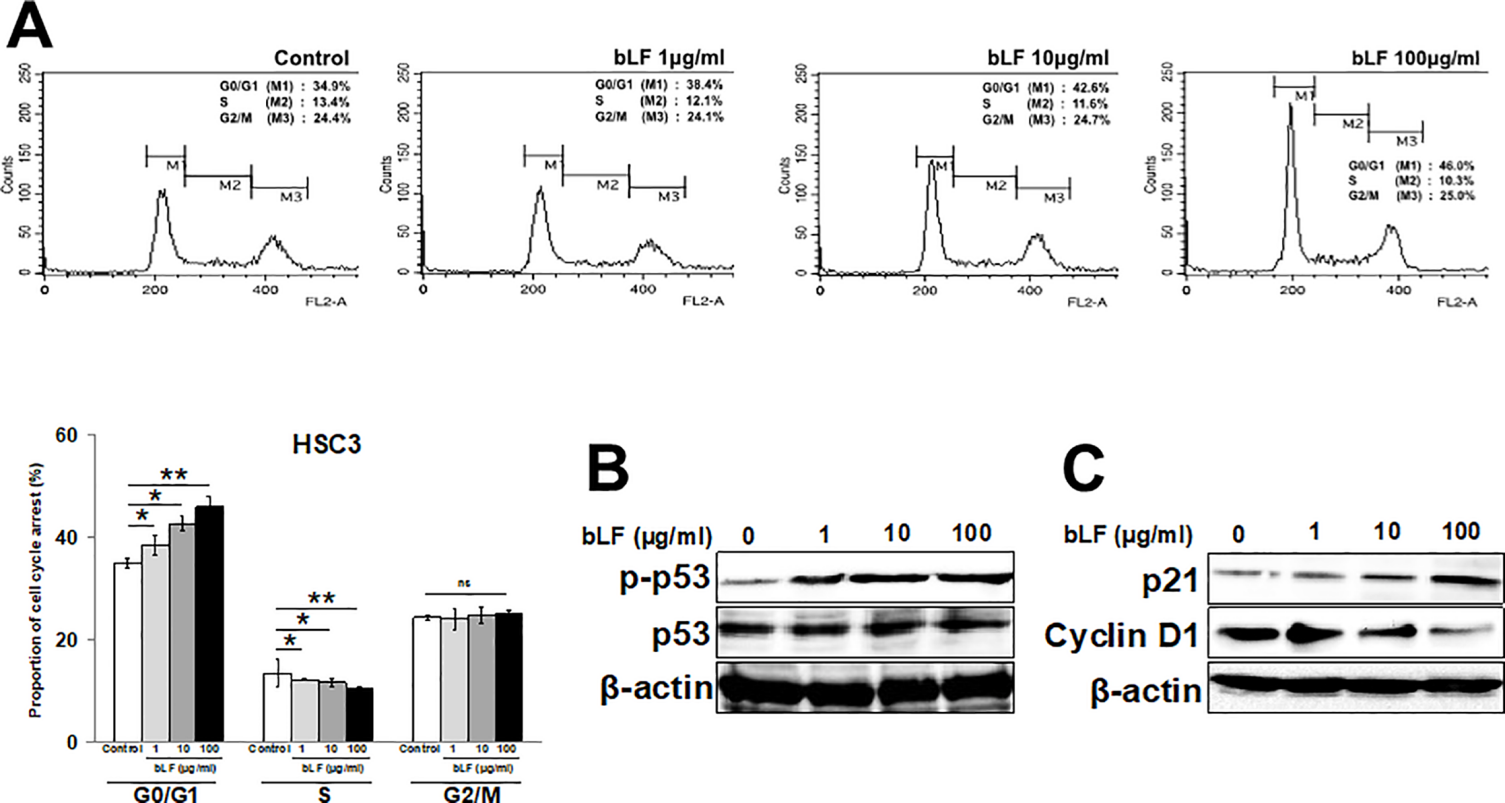
Bovine lactoferrin induced G1/S phase arrest, activation of p53, and regulated cell cycle-related proteins in HSC3 cells. HSC3 cells were treated with various concentrations of bLF (0 μg/ml, 1 μg/ml, 10 μg/ml, and 100 μg/ml). After 48 h, cell cycle analysis was done using flow cytometry. (**A**) bLF induced significant increase in cell population in G0/G1 phase in a dose-dependent manner suggesting the inhibition of G1/S phase transition. Bar graphs are representative for the same. Data were shown as the mean ± S.D; * p < 0.05, ** p < 0.01 *vs* control (0 μg/ml of bLF). (**B**) Expression of p-p53 and total p53 was analyzed 48 h post bLF treatment using western blotting. bLF induced activation of p53 in a dose-dependent manner. (**C**) bLF downregulates expression of Cyclin D1 whereas it enhances p21 expression as determined by western blots. β-actin was used as a loading control. Images are representative of three independent experiments (n = 3).

Since bLF significantly inhibited the cell growth of HSC3 cells, next we checked the effect of bLF on cell cycle progression in HSC3. Cells were treated with bLF (1, 10, and 100 μg/ml) for 48 h, and propidium iodide (PI) was used for cell cycle analysis by flow cytometry. HSC3 cells treated with bLF provoked significant G0/G1 phase arrest in HSC3 cells (38.4%, 42.6%, and 46.0% respectively) as compared to control (34.9%). The arrest in G0/G1 phase led to a significant drop in S-phase cell population from 13.4% in control to 12.1%, 11.6%, and 10.3% in bLF treated HSC3 cells (Fig 2A). These observations indicated that bLF induces cell cycle arrest at G1/S transition.

To further clarify the mechanism involved in bLF-induced cell cycle arrest, we next examined the inhibitory effects of bLF on cell cycle-related molecules. p53, a tumor suppressor protein, is potentially correlated with cell cycle in OSCC [29]. Here, we found that, bLF upregulated p-p53 levels in HSC3 cells in a dose-dependent manner (Fig 2B). Moreover, the accumulation of the cyclin-dependent kinase inhibitor p21, which mediates cellular senescence of HSC3, was positively correlated with bLF treatment (Fig 2C). Reversely, bLF diminished the expression of cyclin D1 (Fig 2C), a regulator protein required for progression through G1 phase of cell cycle. In contrast, we observed that bLF neither suppressed p-p53 and cyclin D1 nor induced accumulation of p21 in RT7 cells (S2A Fig). Our findings indicated that bLF regulates cell proliferation through G1/S cell cycle arrest via modulation of cell cycle-related genes in OSCC but not in normal keretinocyte.

### Bovine lactoferrin induced apoptosis in OSCC cells

**Fig 3:**
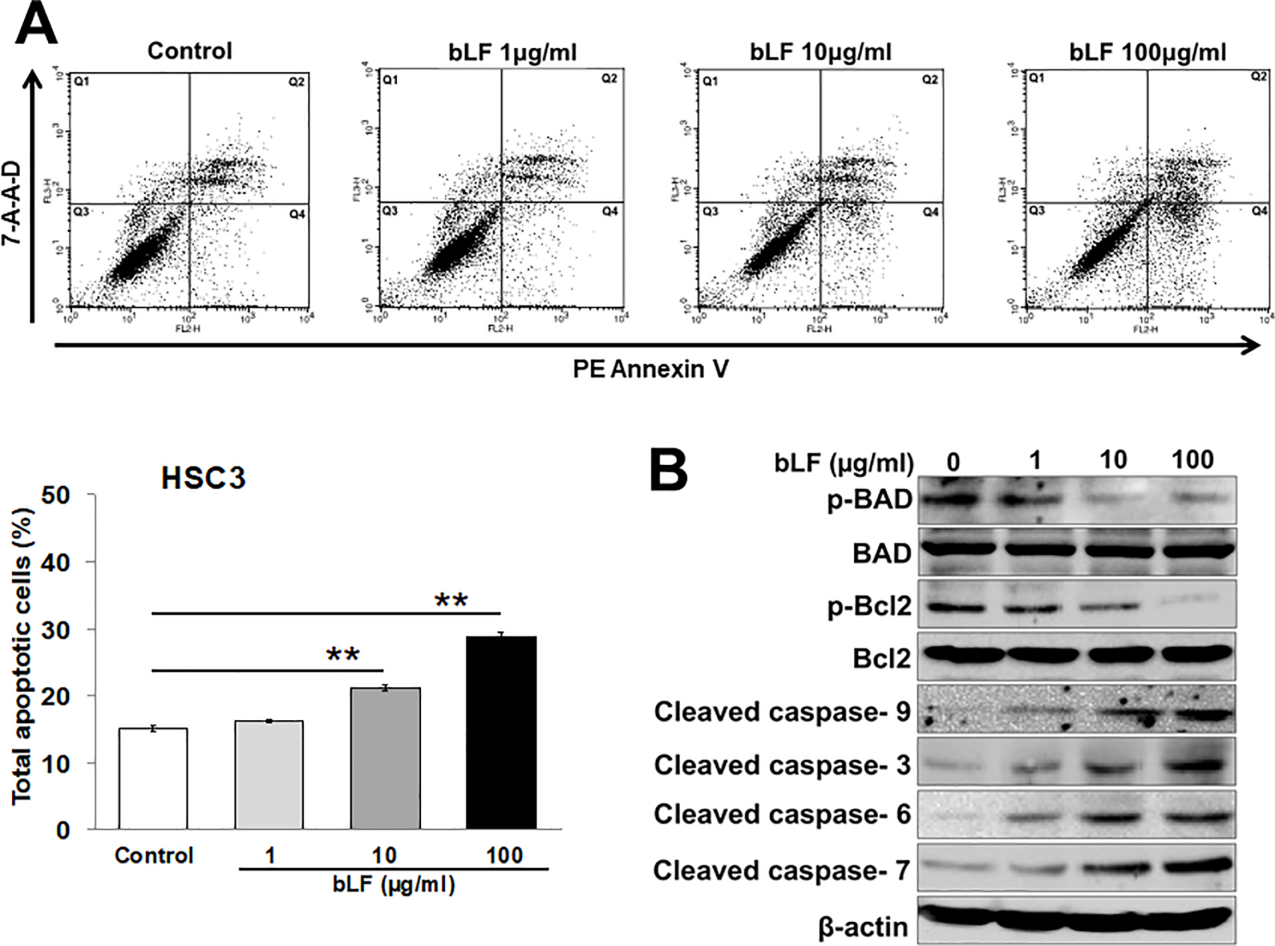
Bovine lactoferrin induced apoptosis in OSCC cells. (**A**) To check the effect of bLF on cell apoptosis, HSC3 cells were incubated with bLF (1 μg/ml, 10 μg/ml, and 100 μg/ml) for 48 h. Percentage of apoptotic cells were then analyzed by flow cytometry using PE Annexin V and 7-AAD. Cell populations were analyzed in four quadrants, Q1: necrotic cells, Q2: late apoptotic cells, Q3: viable cells, and Q4: early apoptotic cells. Total apoptotic cells were measured as the sum of early and late apoptotic cells. A dose-dependent increase in apoptotic cell number was observed after bLF treatment. Data were shown as the mean ± S.D (n = 3); * p < 0.05, ** p < 0.01 vs control. (**B**) HSC3 cells were treated with or without bLF (1, 10, and 100 μg/ml) for 48 h. Expressions of apoptosis-related genes were analyzed by western blotting. bLF treatment inhibited the phosphorylation of BAD and Bcl2 in HSC3 cells whereas expression of cleaved caspases 9, 3, 6, and 7 was upregulated. β-actin was used as a loading control. Data are representative of 3 independent experiments (n = 3).

To further assess the apoptosis-induced growth inhibition of OSCC, HSC3 cells were treated with variable concentration of bLF for 48 h. Cells were then stained with PE-Annexin V and 7-AAD, and analyzed via flow cytometry. Results showed a significant increase in apoptotic cell population in HSC3-treated cells (21.18% at 10 μg/ml of bLF; p = 0.0037 and 28.81% at 100 μg/ml of bLF; p = 0.0007), as compared to that in the control group (15.11%) (Fig 3A). These results suggested that apoptosis is also involved in bLF-mediated growth inhibition of HSC3 cells.

To understand the mechanism of bLF-induced apoptosis, protein expression of Bcl2 family members, such as p-Bad and p-Bcl2 was determined at 48 h post bLF treatment. Western blot analysis indicated that bLF significantly downregulated the protein levels of p-Bad and p-Bcl2 (Fig 3B). In contrast, bLF treatment activated caspase-cascade in HSC3 cells. The protein levels of cleaved caspases 9, 3, 6, and 7 were dramatically increased in bLF treated cells (Fig 3B) as compared to control cells. However, the suppression of Bcl2 family members and the induction of caspase-cascade by bLF were not observed in RT7 cells (S2B Fig). These results proposed the involvement of bLF in apoptotic regulation in OSCC cells through mitochondrial modulation.

### Bovine lactoferrin suppressed the phosphorylation of growth and survival related kinase p65 and Akt in OSCC cells

**Fig 4:**
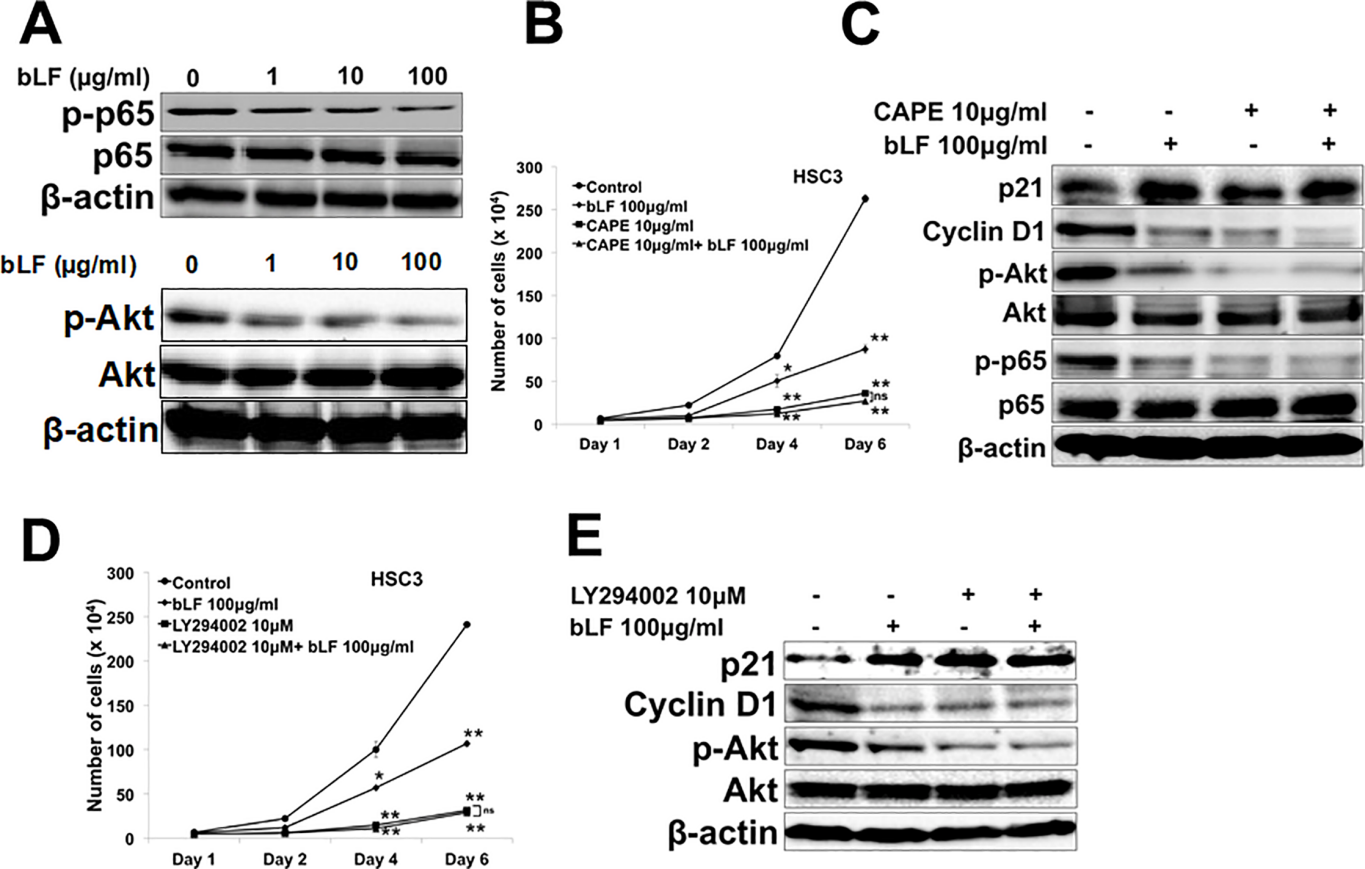
Bovine lactoferrin suppressed the phosphorylation of growth and survival related kinase NF-kB p65 and Akt in HSC3 cells. HSC3 cells were treated with different concentrations of bLF. (**A**) After 48 h, bLF inhibited the activation of growth and survival related kinase p65 and Akt in HSC3 cells. (**B**) Number of HSC3 cells treated with and without CAPE 10μg/ml and bLF were counted. (**C**) G1/S cell cycle arrest related proteins were observed using western blot. (**D**) Proliferation assay were conducted using HSC3 cells under treatment of LY294002 (10 μM) and bLF (100 μg/ml). Cells were counted on day 1, 2, 4, and 6. (**E**) G1/S cell cycle related molecules were examined using western blot after 48 h stimulations of LY294002 and bLF. β-actin was used as loading control. Image is representative of 3 independent experiments (n = 3).

The attenuation of kinase-related signals plays a critical role in the cell growth inhibition. Depletion of NF-kB and Akt arrest cell cycle progression via dysregulation of the cell cycle transitions at G1/S phase [30], and induces apoptosis [31]. Here, we investigated the effects of bLF on p65 and Akt signaling pathways in HSC3 cells. We found that after 48 h of treatment, bLF dramatically downregulated p-p65 and p-Akt expressions in a dose-dependent manner (Fig 4A). To confirm the importance of p65 and Akt involving in growth and cell cycle arrest in HSC3 cells, the use of a specific inhibitors of NF-kB (CAPE; 10μg/ml) and PI3K/Akt (LY294002; 10μM) significantly attenuated the number of HSC3 cells and suppressed G1/S cell cycle related molecules (Fig 4B, 4C, 4D and 4E). These results suggest that NF-kB/Akt is a critical molecule in the inhibitory effect of bLF on cell cycle progression and modulation of apoptosis in OSCC cells. However, the decrease of the p-p65 and p-Akt by bLF were not observed in normal keratinocyte cells RT7 (S3 Fig).

To explain the selective effects of bLF on cells, HSC3 and RT7 were treated with bLF for 48 h. The results showed that levels of phosphorylated Akt were higher in OSCC cells (HSC3) than normal mucosal cells (RT7). bLF significantly inhibited p-Akt levels in HSC3 cells but not in RT7 (S3 Fig). A recent report stated that activation of epidermal growth factor receptor (EGFR)/Akt/mTOR axis acts as a positive feedback for constitutive upregulation of NF-κB subunit p65 in head and neck squamous cell carcinoma [32]. Our study revealed that bLF significantly suppressed p-p65 expression in HSC3 cells without altering the p-p65 status in normal RT7 cells (S3 Fig). These observations suggested the involvement of NF-κB/Akt positive feedback regulation in OSCC.

### Bovine lactoferrin reduced phosphorylation of mTOR and S6K expressions in HSC3 cells

**Fig 5:**
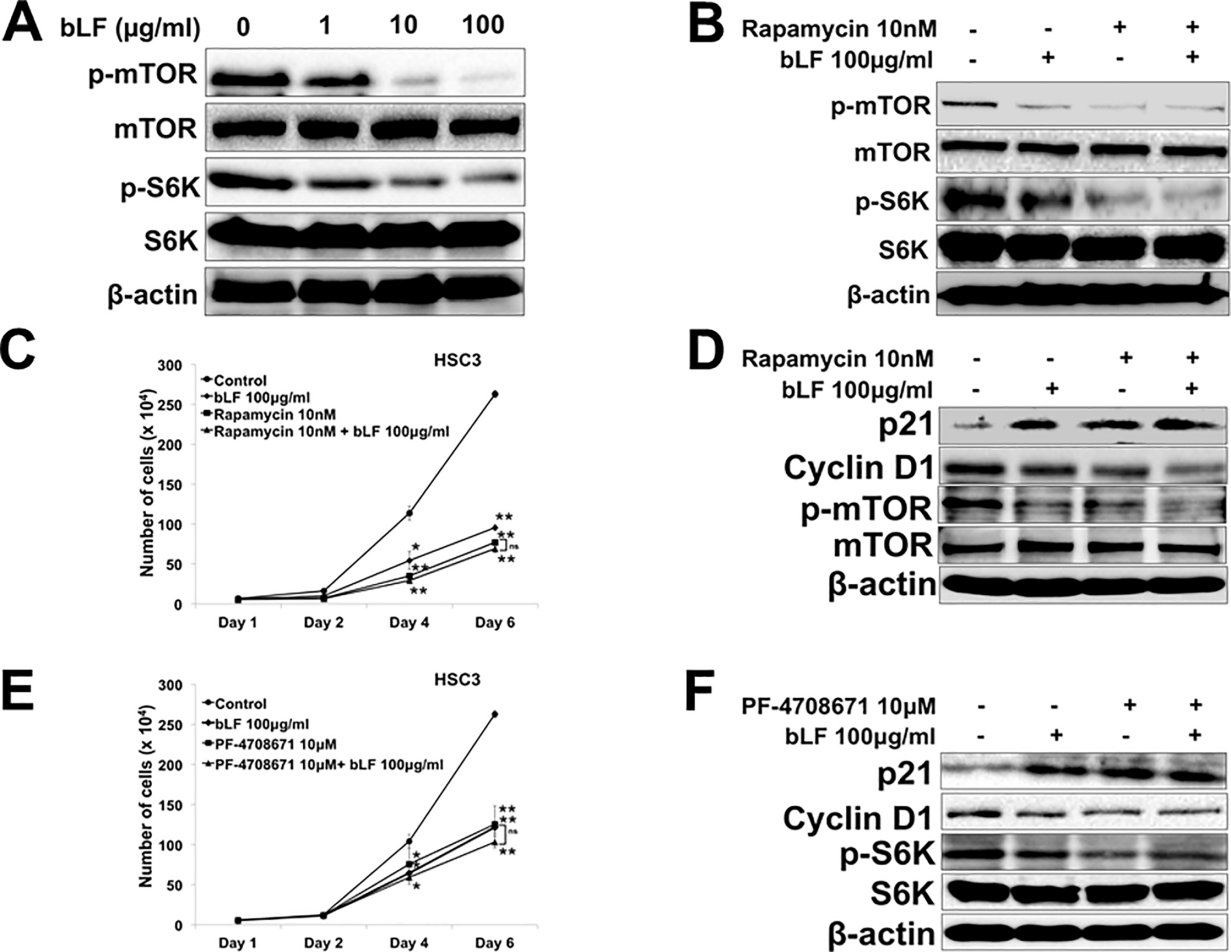
Bovine lactoferrin inhibited expression of mTOR and S6K pathway thereby suppressed growth in HSC3 cells. HSC3 cells were treated with (1 μg/ml, 10 μg/ml, and 100 μg/ml) or without bLF. (**A**) Protein levels of p-mTOR and p-S6K were investigated by western blot after 48 h of stimulations. Expression of phosphorylated mTOR and S6K was decreased after bLF treatment. (**B**) To check the combinatorial effect of bLF and the inhibitor of mTOR, rapamycin, HSC3 cells were treated with 10 nM of rapamycin along with 100 μg/ml of bLF. No additive change was observed on p-mTOR and p-S6K expressions as shown by western blot. (**C**) Number of HSC3 cells was counted on day 1, 2, 4, and 6 with and without rapamycin and bLF. No additional effects on growth of cells in rapamycin and its combination. (**D**) G1/S cell cycle related molecules were investigated using western blot after 48 h of stimulations. Either rapamycin (10 nM) or its combination with bLF accumulated p21 expression but suppressed cyclin D1. (**E**) The growth of HSC3 cells was investigated under treatments of PF-4708671 (10 μM) and bLF 100 μg/ml. PF-4708671 remarkably attenuated number of HSC3 cells. There were no differences in number of cells between PF-4708671 and PF-4708671 with bLF. (**F**) Protein expression of p21 and cyclin D1 were examined using PF-4708671. PF-4708671 induced G1/S cell cycle arrest in HSC3 cells whereas no additive changes in the expressions p21 and cyclin D1 between PF-4708671 and its combination with bLF. β-actin was used as a loading control. The blots represent 3 independent experiments.

The expression of mTOR, which is a key regulator in cell survival, was correlated with tumor stages in clinical-pathological study, whereas the suppression of mTOR and its downstream S6K reduced growth in OSCC cells [33]. Thus, we aimed to elucidate the involvement of bLF on the mTOR/S6K pathway. HSC3 cells were treated with bLF for 48 h and expressions of mTOR and S6K were analyzed by western blotting. bLF downregulated the expression of p-mTOR and p-S6K in a dose-dependent manner (Fig 5A); suggesting that inhibitory effect of bLF on HSC3 cell proliferation could be via inactivation of mTOR/S6K pathway. Interestingly, bLF did not induce any significant changes of p-mTOR and p-S6K expressions in RT7 cells (S4 Fig).

Furthermore, to check the combinatorial effect of rapamycin (an inhibitor of mTOR), PF-4708671 (a specific inhibitor of S6K) and bLF, HSC3 cells were treated with rapamycin (10 nM), PF-4708671 (10 μM), and bLF (100 μg/ml) for 48 h. However, co-treatment of rapamycin, PF-4708671, and bLF did not show any additive changes on expressions of p-mTOR and p-S6K (Fig 5B), complementary effects on number of cells (Fig 5C and 5E), or cell cycle arrest in G1/S of HSC3 (Fig 5D and 5F). These results suggested the novel function of bLF as inhibitor of mTOR/S6K pathway in OSCC.

### Bovine lactoferrin attenuated activation of JAK2/STAT3 pathway through SOCS3 mediated negative feedback modulation

**Fig 6:**
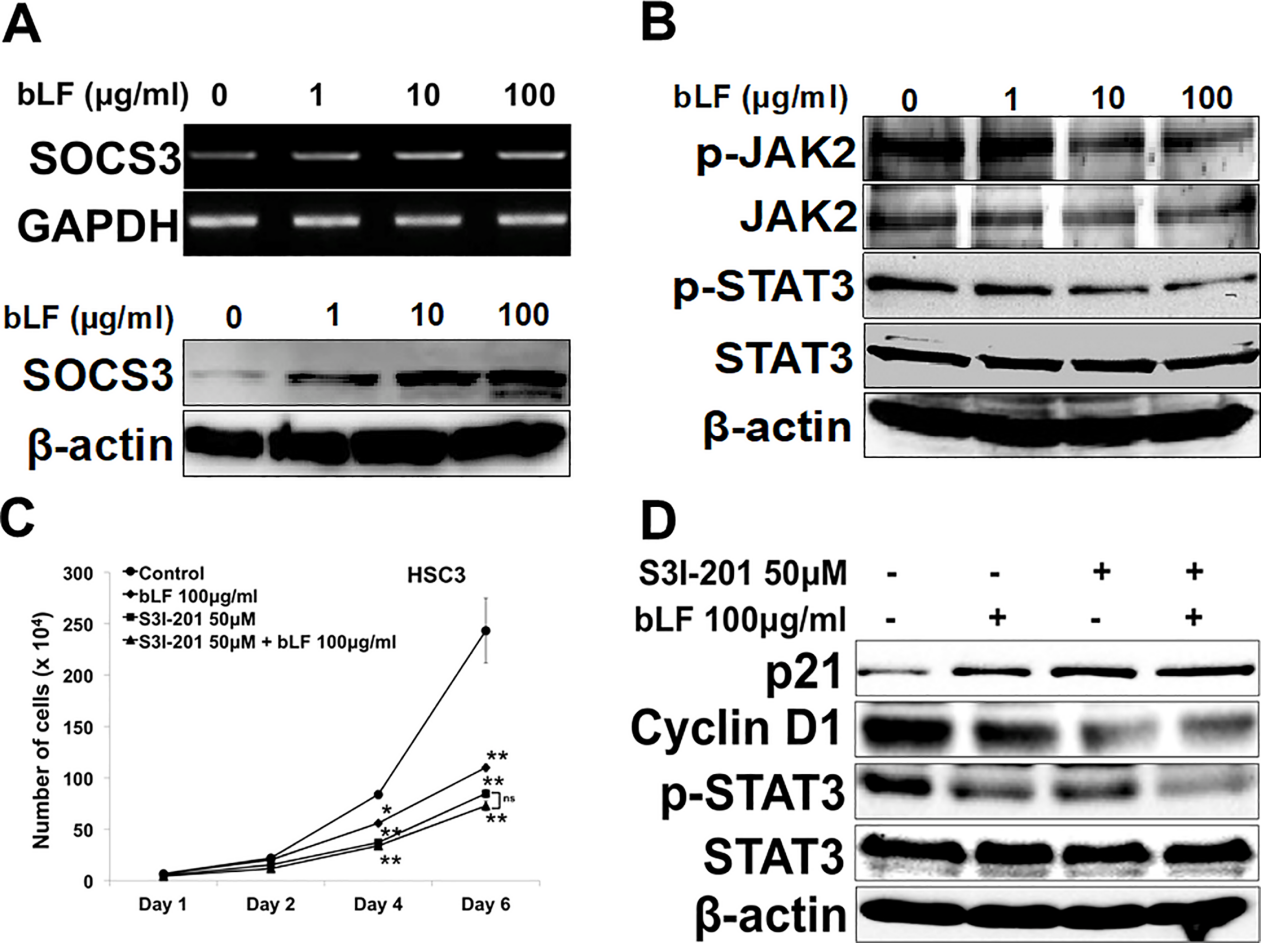
Bovine lactoferrin induced expression of SOCS3 to attenuate JAK2/STAT3 activation involving in inhibition of growth OSCC cells. HSC3 cells were treated with bLF (1 μg/ml, 10 μg/ml, and 100 μg/ml) for 24 h for mRNA and 48 h for protein expressions. (**A**) Expression of SOCS3 was found to be enhanced after bLF treatment checked by RT-PCR and western blot. (**B**) Protein levels of p-JAK2 and p-STAT3 were examined by western blot. bLF inhibited the activation of JAK/STAT3 pathway in a dose-dependent manner. (**C**) Number of HSC3 cells were counted under presence or absence of S3I-201 (50 μM) and bLF (100 μg/ml). Either S3I-201 or bLF-S3I-201 remarkably reduced number of HSC3 cells. On the other hand, no significant suppressive effect of bLF on HSC3 S3I-201 treated cells was observed. (**D**) G1/S cell cycle related molecules were analyzed by western blot in S3I-201 and S3I-201-bLF stimulated HSC3 cells. There were no significant differences in expression of p21 and cyclin D1 in HSC3 cells with S3I-201 and its combination groups. GAPDH and β-actin were used as a loading control. Images are representative of 3 independent experiments (n = 3).

Activation of suppressor of cytokine signaling 3 (SOCS3), a negative regulator of STAT3 signaling, inhibits STAT3 signaling pathway to suppress the proliferation of OSCC [34]. Therefore, we investigated the expressions of SOCS3, p-JAK2, and p-STAT3 in HSC3 cells after bLF treatment. The RT-PCR and immunoblotting results showed that bLF markedly enhanced the expression of SOCS3 both in mRNA and protein levels (Fig 6A) in HSC3 cells but not in RT7 cells (S5 Fig). Interestingly, bLF significantly inhibited p-JAK2 and p-STAT3 levels in HSC3 cells (Fig 6B). To further elucidate the mechanism in which bLF inhibited growth and induced G1/S cell cycle arrest via STAT3 in HSC3, the proliferation assay showed that bLF did not insert additive effects on number of cells and expressions of p21 and cyclin D1 of S3I-201 treated HSC3 cells (Fig 6C and 6D) indicating bLF involved in the inhibition of growth and G1/S cell cycle arrest in OSCC cells through STAT3 pathway. However, these inhibitory effects on JAK2/STAT3 pathway did not observe in RT7 (S5A and S5B Fig). These findings suggested that bLF could suppress OSCC proliferation via SOCS3 mediated inhibition of JAK2/STAT3 signaling pathway.

## Discussion

Recently prevention or treatment of cancer through natural products and dietary interventions has received an increasing interest. Natural anticancer products are safe and have entered clinical trials [35,36]. Numerous studies about the effects of lactoferrin (LF) using human lactoterrin (hLF) and bovine lactoferrin (bLF) on growth of various cancer types were conducted [10, 14, 16– 21, 37]. Moreover, various *in vitro* and *in vivo* investigations [13,38] proved that bLF, a natural anticancer agent, reduces cancer risks by suppressing tumor growth; and has been approved by the European Food Safety Authority, Drug Administration (USA), and the Therapeutic Goods Administration (Australia) [39,40]. Its multi-functional properties render bLF a remarkable impact against cancers including OSCC. To elucidate mechanisms of inhibitory effects of LF on cancer cells, we conducted preliminary investigation using hLF and bLF on OSCC. Our data showed that bLF strongly reduced the proliferation of OSCC cells than hLF (S1 Fig). Hence bLF was selected as a candidate for our current study. In addition, our further results revealed that bLF has anti-proliferative and apoptotic effects on OSCC.

It is well accepted that p53 functions as a mediator to promote cell cycle arrest and apoptosis [41]. Recent studies have shown that bLF-mediated induction of p53 promotes cell cycle arrest in G1/S phase [15] and apoptosis through activation of caspases [13] in different cancers. Phosphorylation of p53 at serine 392 has particularly been reported to be a common and integral event, which blocks the degradation of p53 and increases its stability in response to diverse stimuli [42]. Our results clearly indicated that bLF potentiated phosphorylation of p53 in HSC3 cells (Fig 2B). Activated p53 induced its downstream protein p21, which functionally inhibited cyclin D1 activity (Fig 2C), and arresting the cell cycle in G0/G1 phase.

Recently, mTOR expression has been reported to have a close relationship with poor prognosis in OSCC patients [43]. Thus targeting mTOR poses a beneficial impact on OSCC therapy. To explore the possible mechanisms of bLF activity on the growth inhibition of OSCC, we investigated mTOR kinase, as it integrates multiple signals and regulates cell survival. Surprisingly, we found that bLF downregulated the expression of p-mTOR and its downstream molecule p-S6K. This result provides a novel and critical role of bLF in inhibition of mTOR pathway, thereby reducing cell proliferation in OSCC.

STAT signaling has been involved in oncogenesis [44–46]. The sustained accumulation of STAT3 is correlated with prognosis in OSCC patients [47]. STAT3 inactivation in OSCC reverses the growth phenotype; therefore, STAT3 is a key molecule for the treatment of OSCC. SOCS3, a negative regulator of JAK2/STAT3 signaling, masks STAT3 signaling by binding to JAKs and promoting their proteasomal degradation through ubiquitination [48–49]. Loss of SOCS3 expression has been documented in OSCC [50]. In current study, we found that at 48 h, bLF significantly upregulated the expression of SOCS3 in HSC3 cells in a dose-dependent manner (Fig 6A). This observation suggests a key role of SOCS3-mediated regulation of JAK/STAT3 signaling in OSCC, similar to previous findings [31,51,52]. Furthermore, SOCS3 reduces cancer growth via direct interaction and subsequent stabilization of p53 resulting in enhancement of p21 in pleural mesothelioma cells [53]. Binding of SOCS3 with p53 and JAK2/STAT3 suggests a cross talk among these molecules. Similarly, bLF mediated elevation of p21 levels in OSCC might be correlated with interaction of SOCS3 and p53. Till date, no study has been done to determine the bLF-induced interaction of SOCS3 and p53 in OSCC; therefore, further investigations are needed to elucidate the possible mechanisms.

**Fig 7:**
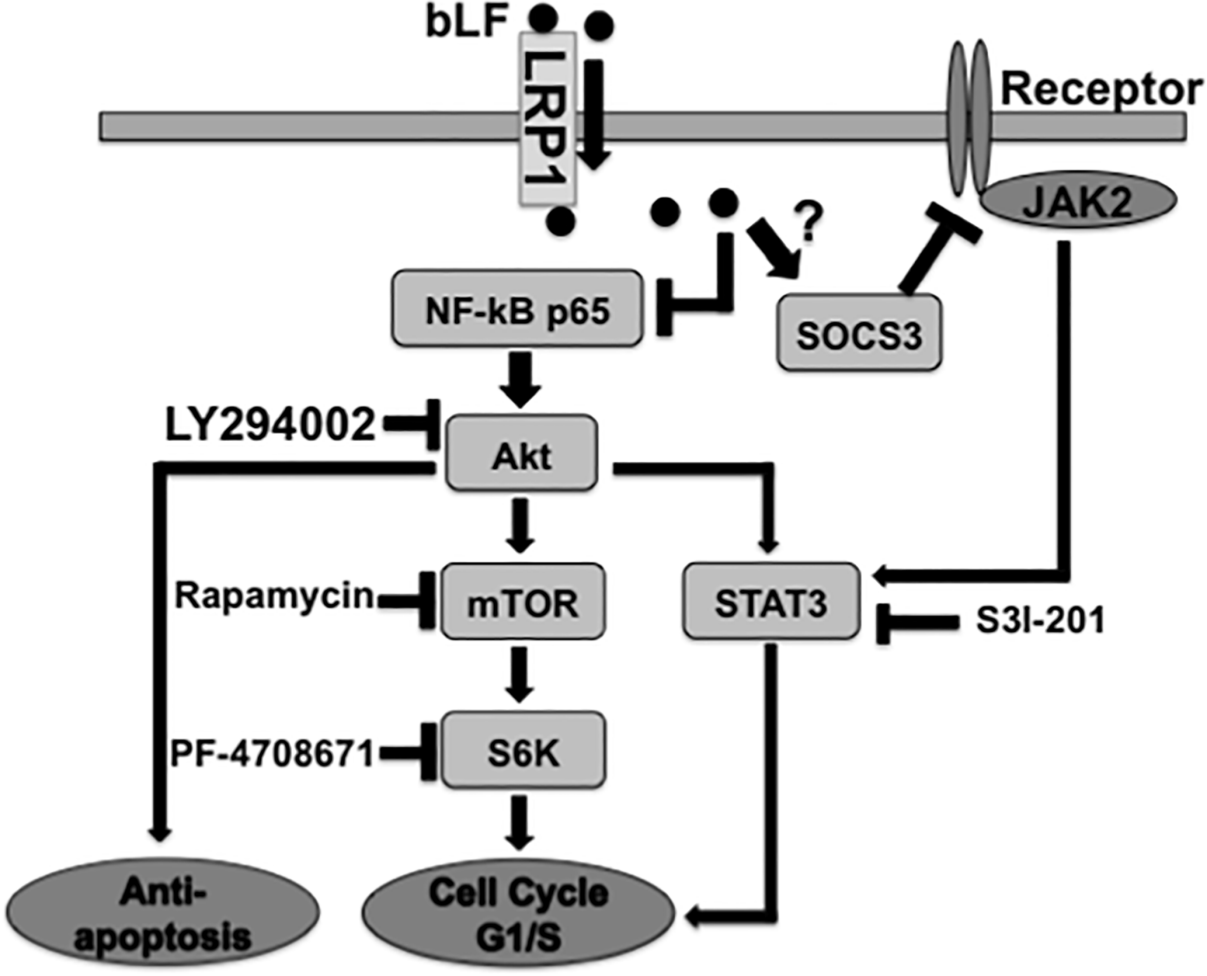
Schema of inhibitory effects of bLF on growth of OSCC cells and its underlying molecular mechanisms. In OSCCs, bLF internalizes into cells through LRP1 and suppressed NF-kB p65/Akt pathways. The attenuation of p65/Akt by bLF may inhibit G1/S cell cycle through the inhibition of downstream signaling pathways including mTOR/S6K and STAT3 and induce apoptosis in OSCC. Moreover, SOCS3, which is a negative regulator of STAT3, is upregulated. The overexpression of of SOCS3 in OSCC by bLF may downregulate JAK2/STAT3 pathway leading to the induction of G1/S cell cycle arrest.

bLF has been demonstrated to increase apoptosis in various cancers by activating caspases such as caspase 9, 3, 7, and 8 [9,18,20,37]. However, the mechanism by which bLF induces apoptosis in OSCC is still not well understood. Our data demonstrated that bLF significantly induced apoptosis in HSC3 cells, as observed by PE Annexin V/ 7-AAD staining (Fig 3A). At molecular level, we found that increased phosphorylation and stabilization of p53 (Fig 2B), decreased levels of anti-apoptotic proteins p-Bad and p-Bcl2, and caspase-cascade activation (Fig 3B) are responsible for bLF-induced apoptosis. Akt pathway is known to contribute in proliferation and apoptotic signaling effects in different cancers [15–17]. In addition, Akt is also reported to activate mTOR and NF-κB activation. Increasing evidences indicated that NF-κB (p65) was highly expressed in OSCC as compared to normal mucosa [54]. In addition, the activated NF-κB provides a positive feedback to regulate EGFR/Akt/mTOR pathway, thereby promoting the cell proliferation in cancers such as HNSCC [32]. Based on our observations, bLF-mediated attenuation of p-p65 and p-Akt in HSC3 cells suppressed phosphorylation of mTOR and S6K, induced cell cycle arrest in G1/S (Figs 4A, 5A, 5D, 5F and 7), and was involved in apoptotic modulation of OSCC.

Recently, our group has shown that bLF is endocytosed through lipoprotein receptor-related protein-1 (LRP1) and bound to endogenous TRAF6 to inhibit TRAF6-dependent activation of the NF-κB signaling pathway [55]. In this study, the existence and the substantial level of LRP1 in SCC of the tongue, various examined OSCC cell lines, and bLF treated HSC3 cells (S6A, S6B and S6C Fig) may explain the possible mechanism of suppressive effects of bLF on NF-kB/Akt signaling and downstream molecules in OSCC (Fig 7). Recently, increasing number of publications focusing on contributive roles of the CXC chemokine receptor 4 (CXCR4) on growth and cell cycle of OSCC and esophageal carcinoma using PI3/Akt were reported [56,57]. In addition, CXCR4 was also demonstrated to be one of receptors of bLF driving for intracellular responses via PI3K/Akt pathway in human keratinocytes HaCaT and human intestinal cells Caco-2 [58]. According to the relationship and functions of CXCR4 and bLF, the study on roles of CXCR4 and its ligand bLF in OSCC cells should be considered for further studies.

In current study, we found that HSC3 cells constitutively expressed activated Akt and p65 (Fig 4A and S3 Fig). bLF treatment downregulated the levels of p-p65 and p-Akt in HSC3 cells, eventually leading to cell growth inhibition and induction of apoptosis via modulation of Akt-related signaling. On the other hand, since RT7 cells have low expression of both p-Akt and p-p65, therefore, bLF could not exert its inhibitory effects (S3 Fig). Based on our further investigation in molecular levels, under bLF treatment no significant changes in p53, caspase cascades, and related molecules of G1/S cell cycle progression were seen in RT7 cells (S2A and S2B Fig). In addition, bLF did not also have any inhibitory effects on regulation of mTOR/S6K pathway, one of an important pathways driving for OSCC cells survival (S4 Fig). Nevertheless, bLF regulated neither SOCS3 nor its negative mediators JAK2/STAT3 in normal keratinocyte RT7 cells (S5A and S5B Fig). These observations suggest that inhibitory action of bLF depends on the status of activated NF-κB, which is reduced by the inhibition of polyubiquitination of endogenous TRAF6 of OSCC. The reduction of NF-kB signaling may further contribute the downregulation of Akt as the result of feedback regulation between NF-kB and Akt. As a consequence, bLF selectively suppresses proliferation in OSCC but not in normal mucosal cells.

Among the various properties of bLF, selective inhibition of cell proliferation in OSCC is an interesting feature. In this study, we showed that bLF imparts various cytological effects to control HSC3 cell proliferation, while showing a very low detrimental effect on normal oral mucosal cells (RT7). However, the mechanism by which bLF suppresses growth of OSCC cells but not normal mucosal cells is still unclear. There are few possibilities to explain this phenomenon. First, possibility includes that binding of bLF with cell surface or intracellular receptors could bring out different functional consequences. Cells may respond differently to bLF based on its extracellular and/or intracellular localization. Previous reports indicated that a big fraction of bLF stayed outside the cells [59], whereas some reports showed bLF internalization by cancer cells [60]. Therefore, the possibility of cellular adjustment of OSCC and normal cells according to bLF-microenvironment cannot be excluded. Depending upon the localization of bLF, similar stimuli may trigger differential expression of proliferation and apoptosis-related targets in OSCC and normal cells. Therefore, the observation of extracellular and intracellular intake of bLF as well as cellular signals might explain the selective effect of bLF on HSC3 cells. Second, it is still unclear how a big molecule like bLF exerts its effects intracellularly in different cell-types. There is a possibility that bLF breaks into small peptides after its internalization especially in cancer cells. The small peptides of bLF may be responsible for the cellular regulation and inhibitory effects on OSCC cells. Thus, it would be interesting to investigate the possible phenomenon of bLF-breakdown in OSCC and normal cells. In short, comprehensive study is needed to explain the specific effects of bLF on OSCC rather than normal mucosa cells both in terms of G1/S cell cycle arrest and caspase-cascade activation.

## Conclusions

In conclusion, bLF regulates cell cycle, cell proliferation, and apoptosis pathways to reduce cellular growth in OSCC and has minimal effects on normal mucosa. To the best of our knowledge, this is the first report showing the effects of bLF on mTOR/S6K pathway inhibition and negative modulation of JAK2/STAT3 signaling via SOCS3. This study could provide a forward direction in the field of lactoferrin research and bLF could be considered as a potential molecule in OSCC prevention and/or therapy.

## Supporting information

S1 Text Supplemental methods. Reagents; Patient specimens; Immunohistochemistry.
(DOCX)

S1 Fig Bovine lactoferrin and human lactoferrin have potential effects on proliferation of HSC3 cells. HSC3 cells were cultured and treated with and without hLF (1, 10, and 100μg/ml) and bLF (1, 10, and 100μg/ml). Number of cells was counted in day 1, 2, 4, and 6. (A) bLF significantly prohibited the cell proliferation of OSCC in a dose-dependent manner. (B) hLF slightly suppressed proliferation of HSC3 cells. Data represented as mean ± S.D; * *p* < 0.05 and ** *p* < 0.01 *vs* control (0 μg/ml of bLF and hLF).
(TIF)

S2 Fig Bovine lactoferrin did not insert any effects on activation of p53, cell cycle-related proteins, and cells induced apoptosis in RT7 cells. RT7 cells were stimulated with and without bLF (1, 10, and 100 μg/ml) for 48 h. Proteins were harvested and analyzed using western blot. (**A**) Expressions of p-p53, p21, and cyclin D1 were investigated. bLF did not show potential effect on regulation of p-p53 and G1/S cell cycle related molecules. (**B**) Expressions of apoptosis-related proteins were observed by western blotting. bLF neither inhibited the phosphorylation of BAD and Bcl2 in RT7 cells nor induced expression of cleaved caspase 9. β-actin was used as a loading control. All western blot experiments were conducted at least 3 times (n = 3).
(TIF)

S3 Fig Bovine lactoferrin suppressed the expression of p-p65 and p-Akt in HSC3 but not in RT7. After 24 h of culture, HSC3 and RT7 cells were treated with bLF for 48 h. Cells were lysed and protein expression was checked by western blot. Expression of cell proliferation related proteins, p-Akt and p-p65 were reduced in HSC3 cell line after bLF treatment; however, bLF did not affect the status of these proteins in normal human oral keratinocyte RT7. β-actin was used as a loading control. All western blot experiments were performed at least 3 times (n = 3).
(TIF)

S4 Fig Bovine lactoferrin did not inhibit mTOR/S6K pathway in RT7 cells. After 48 h of bLF (1, 10, and 100 μg/ml) treatment, RT7 cells were collected and extracted for proteins. Phosphorylation of mTOR and p-S6K were detected by western blot. bLF did decrease expressions of p-mTOR and p-S6K. β-actin was used as a positive loading. Experiments were observed at least 3 times (n = 3).
(TIF)

S5 Fig Bovine lactoferrin did not increase expression of SOCS3 to decrease JAK2/STAT3 activation in RT7 cells. Stimulated RT7 cells under presence and absence of bLF (1, 10, and 100 μg/ml) for 48 h were collected and investigated using western blot. (**A**) Protein expression of SOCS3 was analyzed. bLF did not elevated the expression of SOCS3 in normal mucosa cells. (**B**) Expression of p-JAK2 and p-STAT3 were observed by western blot. bLF did not inhibit the activation of JAK/STAT3 pathway in RT7 cells. β-actin was used as a loading control. All experiments were conducted at least 3 times (n = 3).
(TIF)

S6 Fig Expression of LRP1 in clinicopathology and expression of LRP1 in HSC3 cells was observed. (A) Expression of LRP1 in OSCC cell lines was checked using RT-PCR and western blot. All examined OSCC cell expressed LRP1. (B) LRP1 expression of 48h bLF (1, 10, and 100 μg/ml) treated HSC3 cells was analyzed using by western blot. bLF did not decrease the expression of LRP1 in HSC3. (**C**) Tongue SCC cases were sectioned and stained with anti-LRP1. LRP1 was positively stained in SCC tissue. β-actin was used as a loading control. All experiments were conducted at least 3 times (n = 3).
(TIF)

## Abbreviations

bLF: bovine lactoferrin
OSCC: oral squamous cell carcinoma
