# Supplementary material for: Molecular mechanism of inhibitory effects of bovine lactoferrin on the growth of oral squamous cell carcinoma
Source: PLoS One. 2018 Jan 30;13(1):e0191683. doi: 10.1371/journal.pone.0191683 (PMC5790278; doi:10.1371/journal.pone.0191683)
Supplement: S1 Text — Reagents; Patient specimens; Immunohistochemistry. (DOCX) [file pone.0191683.s001.docx]

# Supplemental methods

## Reagents

Human lactoferrin (hLF) was purchased from Sigma-Aldrich (L6793; MO, USA).

# Patient specimens

Twelve cases of tongue SCC were retrieved from the pathological files of Hiroshima University Hospital, Japan. All cases involved first operation specimens, showing T1N0M0 or T2N0M0. Clinical details including the patient age, sex, tumor location and tumor size were gathered from surgical records of the patients ( 8 males and 4 females; age 5.94 ± 14.9 years (35 - 77 years old); 8 T1N0M0 and 4 T2N0M0). The study was approved by the ethnical committee of Hiroshima University (Permit Number: E-991).

# Immunohistochemistry

Unstained 4.5-µm sections were de-paraffinized and rehydrated by routine histological techniques. Endogenous peroxidase activity was blocked with 0.3% H_2_O_2_ in methanol for 30 minutes. The sections were then incubated with protein block serum-free solution (DAKO, Japan) for 10 minutes. LRP1 antibody (ab92544; 1:150; abcam) was diluted in sterile PBS and incubated overnight at 4°C. The sections were incubated with labeled polymer-HRP-anti-rabbit (DAKO) for 1 hour at room temperature. The color was developed with 0.025% 3,3’-diaminobenzidine tetrahydrochloride in Tris-HCl buffer plus hydrogen peroxide (DAB; DAKO).
