# Supplementary figures and images for: Molecular mechanism of inhibitory effects of bovine lactoferrin on the growth of oral squamous cell carcinoma
Source: PLoS One. 2018 Jan 30;13(1):e0191683. doi: 10.1371/journal.pone.0191683 (PMC5790278; doi:10.1371/journal.pone.0191683)

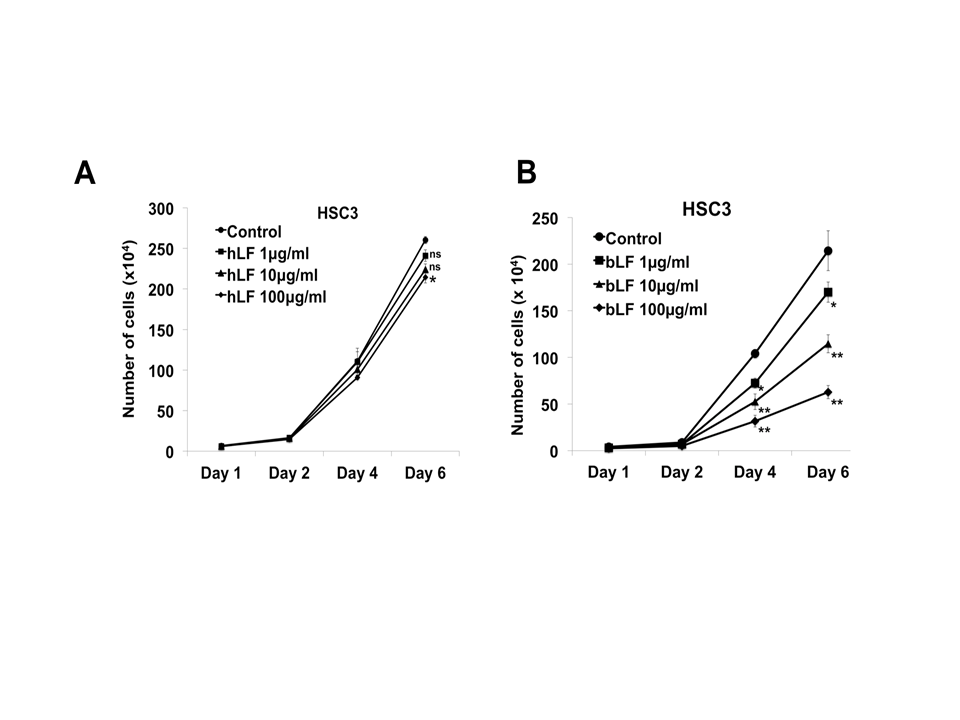

Supplement: S1 Fig — HSC3 cells were cultured and treated with and without hLF (1, 10, and 100μg/ml) and bLF (1, 10, and 100μg/ml). Number of cells was counted in day 1, 2, 4, and 6. (A) bLF significantly prohibited the cell proliferation of OSCC in a dose-dependent manner. (B) hLF slightly suppressed proliferation of HSC3 cells. Data represented as mean ± S.D; * p < 0.05 and ** p < 0.01 vs control (0 μg/ml of bLF and hLF). (TIF) [file pone.0191683.s002.TIF]

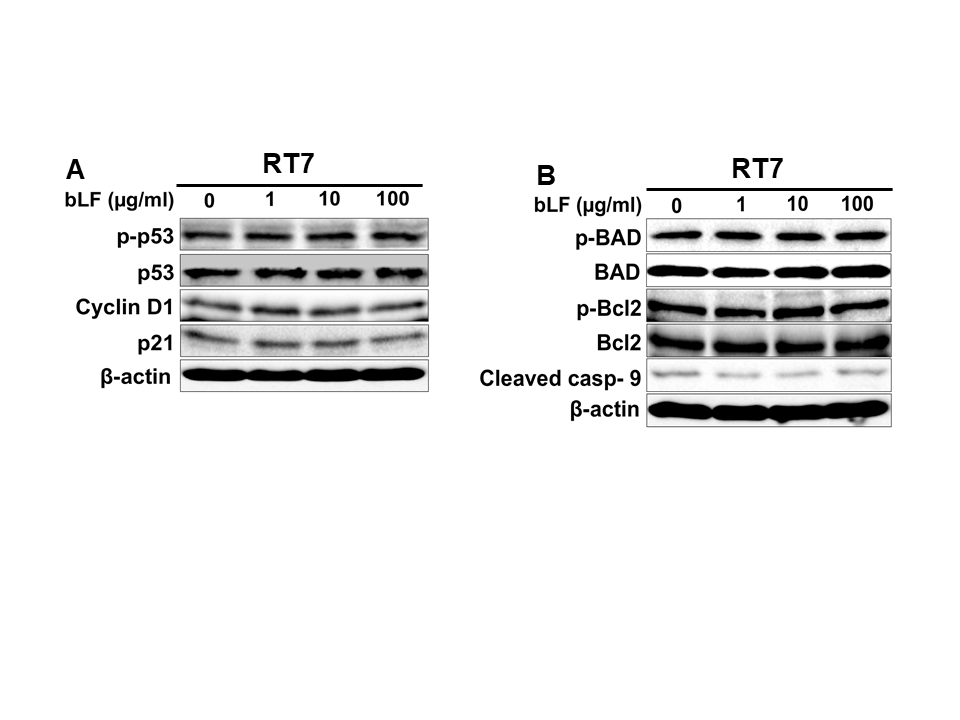

Supplement: S2 Fig — RT7 cells were stimulated with and without bLF (1, 10, and 100 μg/ml) for 48 h. Proteins were harvested and analyzed using western blot. (A) Expressions of p-p53, p21, and cyclin D1 were investigated. bLF did not show potential effect on regulation of p-p53 and G1/S cell cycle related molecules. (B) Expressions of apoptosis-related proteins were observed by western blotting. bLF neither inhibited the phosphorylation of BAD and Bcl2 in RT7 cells nor induced expression of cleaved caspase 9. β-actin was used as a loading control. All western blot experiments were conducted at least 3 times (n = 3). (TIF) [file pone.0191683.s003.TIF]

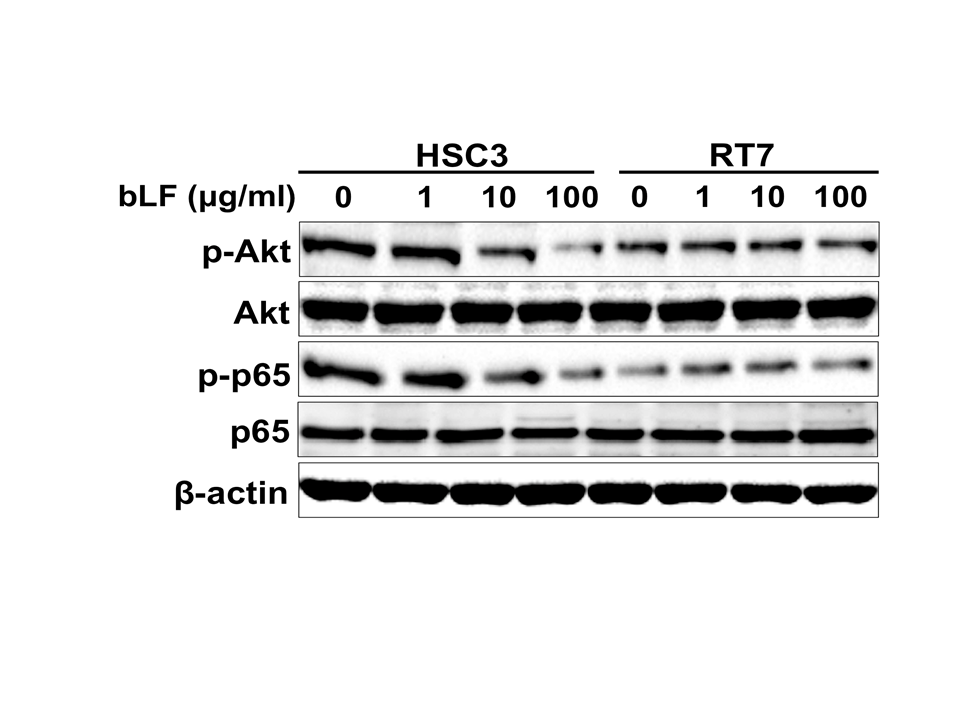

Supplement: S3 Fig — After 24 h of culture, HSC3 and RT7 cells were treated with bLF for 48 h. Cells were lysed and protein expression was checked by western blot. Expression of cell proliferation related proteins, p-Akt and p-p65 were reduced in HSC3 cell line after bLF treatment; however, bLF did not affect the status of these proteins in normal human oral keratinocyte RT7. β-actin was used as a loading control. All western blot experiments were performed at least 3 times (n = 3). (TIF) [file pone.0191683.s004.TIF]

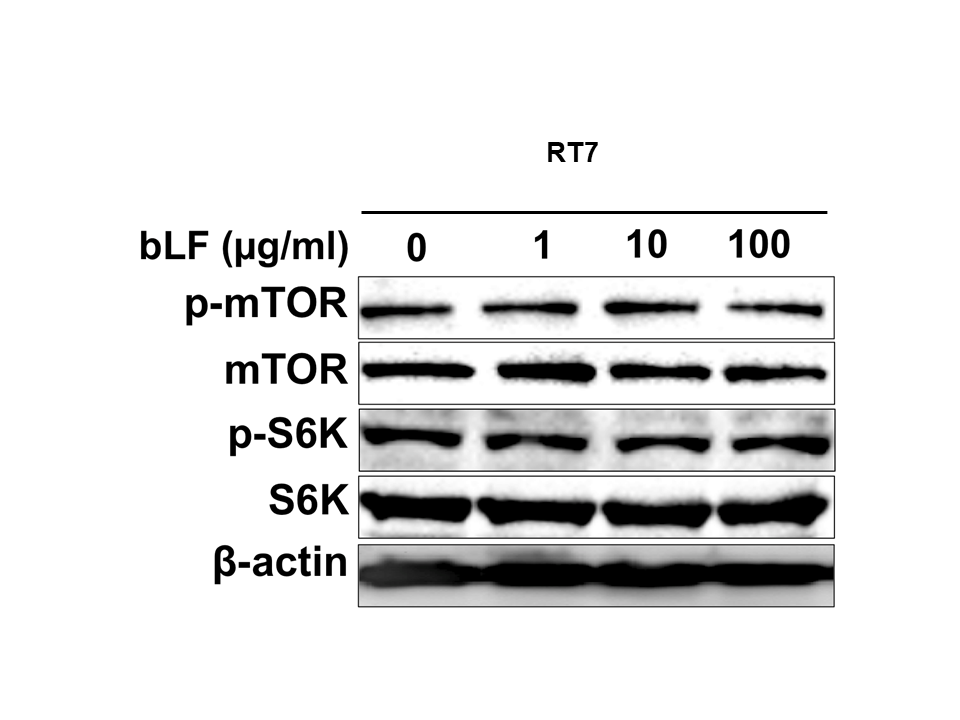

Supplement: S4 Fig — After 48 h of bLF (1, 10, and 100 μg/ml) treatment, RT7 cells were collected and extracted for proteins. Phosphorylation of mTOR and p-S6K were detected by western blot. bLF did decrease expressions of p-mTOR and p-S6K. β-actin was used as a positive loading. Experiments were observed at least 3 times (n = 3). (TIF) [file pone.0191683.s005.TIF]

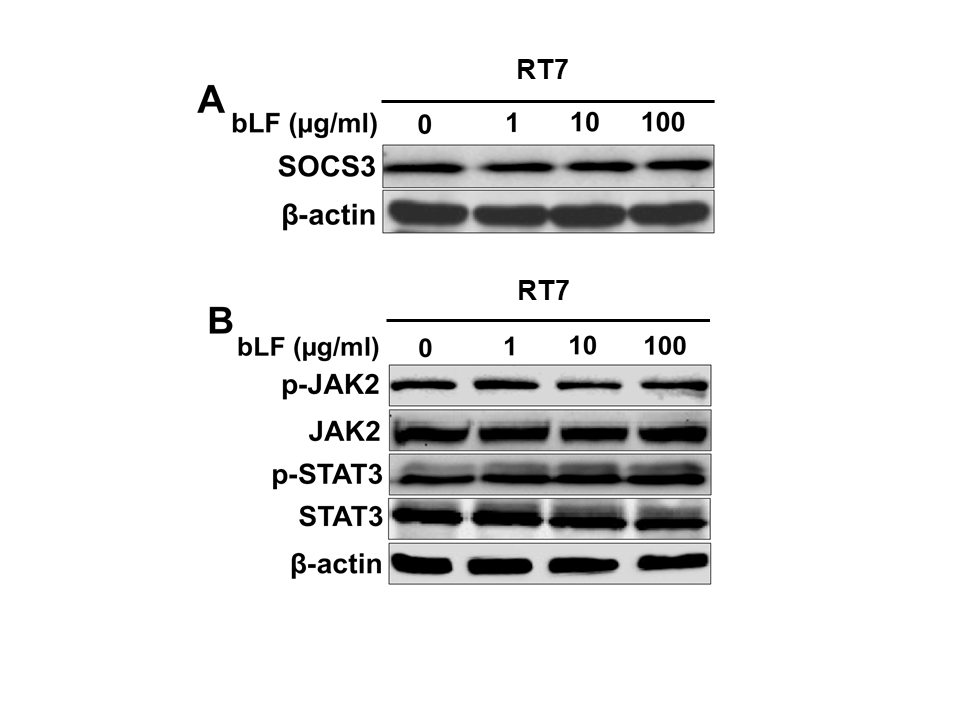

Supplement: S5 Fig — Stimulated RT7 cells under presence and absence of bLF (1, 10, and 100 μg/ml) for 48 h were collected and investigated using western blot. (A) Protein expression of SOCS3 was analyzed. bLF did not elevated the expression of SOCS3 in normal mucosa cells. (B) Expression of p-JAK2 and p-STAT3 were observed by western blot. bLF did not inhibit the activation of JAK/STAT3 pathway in RT7 cells. β-actin was used as a loading control. All experiments were conducted at least 3 times (n = 3). (TIF) [file pone.0191683.s006.TIF]

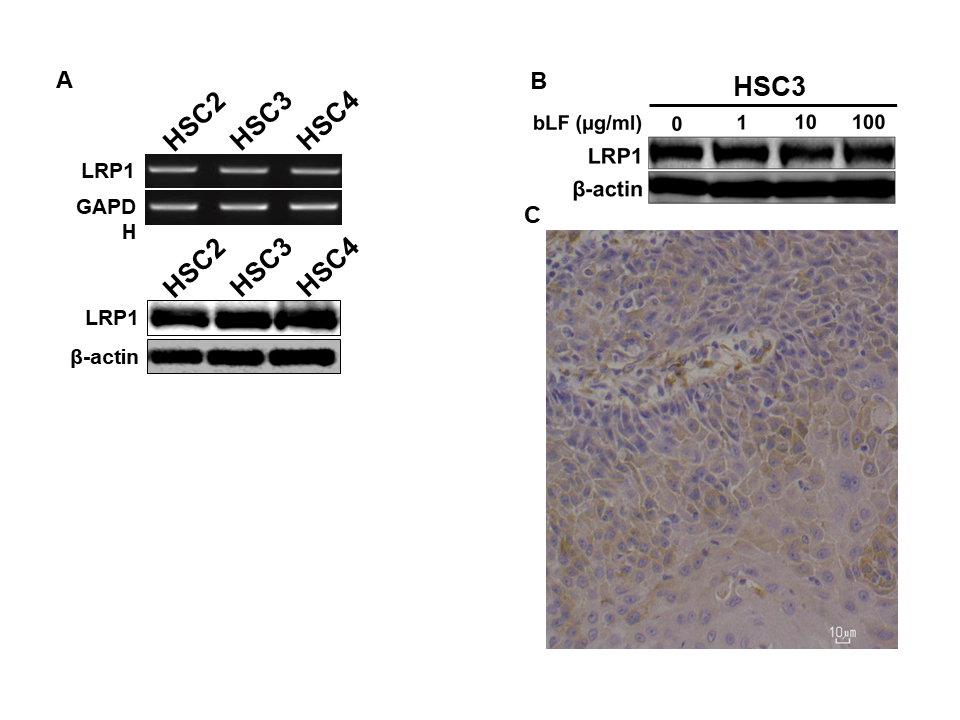

Supplement: S6 Fig — (A) Expression of LRP1 in OSCC cell lines was checked using RT-PCR and western blot. All examined OSCC cell expressed LRP1. (B) LRP1 expression of 48h bLF (1, 10, and 100 μg/ml) treated HSC3 cells was analyzed using by western blot. bLF did not decrease the expression of LRP1 in HSC3. (C) Tongue SCC cases were sectioned and stained with anti-LRP1. LRP1 was positively stained in SCC tissue. β-actin was used as a loading control. All experiments were conducted at least 3 times (n = 3). (TIF) [file pone.0191683.s007.TIF]
